# Beyond ethics and aesthetics: perceived lay theory violations and agency disruption in AI-generated art

**DOI:** 10.3389/fpsyg.2026.1716814

**Published:** 2026-06-22

**Authors:** Melissa J. Dolese

**Affiliations:** Department of Social Science, University of Alaska Southeast, Juneau, AK, United States

**Keywords:** agency, AI, art, authenticity, creativity, embodiment, emotion, perception

## Abstract

The use of AI to generate art has sparked responses ranging from fascination to anger and resistance, raising fundamental questions about the nature of creativity and human artistic identity. This paper argues that the devaluation of AI-generated work stems in part from disruptions to culturally ingrained lay theories of creativity. These intuitive frameworks link artistic value to human creative performance, particularly the perceived “spark” of embodied insight and a coherent history of generative human agency. Unlike traditional creative processes, AI is perceived as proceduralizing the associative processes underlying creative insight, producing artifacts in which the link between the creative performance and the artistic outcome is no longer readily reconstructible. This shift generates a psychological gap: observers may recognize the aesthetic quality of an artifact while struggling to anchor it to a coherent human generative process. Drawing on frameworks of agency mapping, artistic communication, and appraisal-based emotion, the analysis shows how AI disrupts the mechanisms that audiences rely on to value and categorize art. Rather than tracking surface cues in the artifact, these mechanisms depend on reconstructing a human creative performance that links intention, process, and outcome into a unified generative history. When this reconstruction fails, artifacts no longer reliably satisfy mechanisms such as magical contagion, the extended self, the artistic design stance, and communicative inference, all of which depend on perceiving a human mind as the originating source of creative action. This structural breakdown elicits two core emotional responses: aesthetic anger, triggered by violations of expectations about human creative authorship, and ontological unease, arising from the simulation of uniquely human creative processes by a non-biological system. The paper further examines organizational framings of AI-generated art—as Tool, Collaborator, or Threat—highlighting how these interpretations attempt to stabilize uncertainty in agency reconstruction. Understanding these cognitive and emotional dynamics clarifies why AI-generated art provokes resistance even when its outputs are aesthetically compelling.

## 1 Introduction: appraisal, agency, and the evaluation of AI-generated art

The emergence of AI-generated art has challenged the cultural assumptions that define the value of art, creating a friction that extends beyond mere technical or legal concerns. While issues such as appropriation, job displacement, and copyright ambiguity remain central (Crawford and Paglen, 2021), they do not fully capture the range of public responses, which fluctuate between curiosity and fascination (Grassini and Koivisto, 2024; Shah, 2023) and persistent resistance toward machine-mediated creativity (Millet et al., 2023).

Observers’ reactions to AI-generated outputs raise fundamental questions about creativity, authorship, and the categorization of art. In a broad cross-disciplinary review, Anantrasirichai and Bull (2022) observed that generative processes had reached a level of sophistication where they mirrored human creative outputs across multiple domains. In music, researchers demonstrated high-fidelity audio synthesis capable of replicating complex harmonic structures (Donahue et al., 2018; Engel et al., 2019), while in literature, Köbis and Mossink (2021) found that observers struggled to distinguish machine-made poetry from human-written verse. Gu et al. (2022) showed that these trends were particularly salient in the visual arts, where diffusion architectures produced high-fidelity synthesis that replicated the stylistic patterns required to elicit evaluative judgements comparable to human art (Chamberlain et al., 2018; Jago, 2019). Although Van Hees et al. (2025) reported that observers could still detect synthetic origins at above-chance levels, they noted that the work’s formal quality remained high across perceptual and compositional features (Elgammal et al., 2017; Gangadharbatla, 2022). When creator labels were absent, Hong and Curran (2019)found that audiences faced significant difficulty in reliably distinguishing these artifacts from human-produced work.

Despite these perceptual similarities, de Rooij (2025) found that a consistent gap emerged between sensory appreciation and perceived value. Even when observers evaluated identical artifacts, knowledge of AI authorship altered judgments of creativity, authenticity, and artistic merit (Hong and Curran, 2019; Ragot et al., 2020). In some cases, observers preferred AI-generated works when their origin was unknown (Van Hees et al., 2025) yet exhibited a subtle but persistent negativity bias when works were labeled as AI-generated (de Rooij, 2025). Identical artifacts were rated as less creative, intentional, and authentic when attributed to machines rather than humans, a phenomenon that Millet et al. (2023), Bellaiche et al. (2023), and Ragot et al. (2020) described as the creator label effect. This bias occurred even when no perceptible differences existed between artifacts.

Related research suggested that AI-labeled works elicited weaker engagement from audiences. For instance, Chamberlain et al. (2018) reported that observers held lower perceived authenticity and creative value for AI-labeled works. Furthermore, participants spent less time attending to them (Zhou and Kawabata, 2023) and attributed less effort and creative value than to identical works labeled as human-made (Horton et al., 2023).

Together, these findings suggest that resistance to AI-generated art is not primarily a reaction to aesthetic quality, but to the perceived origin of the artifact. Such responses may reflect broader anthropocentric motivations to preserve the distinctiveness of human creativity (Millet et al., 2023). When observers cannot easily map an artifact onto a coherent model of human generative activity, evaluations shift, often resulting in devaluation and resistance. de Rooij (2025) argued that this resistance is consistent with interpretations that located the effect primarily in higher-level interpretive systems concerned with authorship and meaning, rather than within basic sensory–motor aesthetic processing.

The present analysis organizes responses to AI-generated art into three broad framings reflected in prior scholarship: AI as a tool extending human creativity and agency (Hertzmann, 2018; McCormack et al., 2019; Trach, 2021); AI as a collaborator participating in a shared creative process while preserving human agency (Epstein et al., 2020; Zylinska, 2020); and AI as a threat, wherein generative systems are described as disrupting human agency and creative labor (Javed et al., 2024; Köbis and Mossink, 2021; Wade, 2023). These framings shape how audiences interpret AI-mediated artworks and engage lay theories of creativity and the psychological processes through which artistic value is typically inferred. Depending on which framing is activated, audiences may accept outputs as extensions of human agency or devalue them as violations of expectations surrounding authorship and intentionality.

Attribution-based theories of emotion provide a framework for understanding the affective consequences of these shifts (Scherer, 2001; Silvia and Brown, 2007; Weiner, 1985). Within these models, researchers proposed that emotional responses arise from appraisals of causal structure, including locus (internal vs. external), stability (temporary vs. enduring), controllability (manageable vs. uncontrollable), and perceived intentionality. When AI systems produce outputs that appear to bypass human generative processes, the inferred locus of causation may shift away from the human agent, perceived controllability may decrease, and the generative process may appear stable and replicable. Such appraisals may be associated with affective responses consistent with anger, reflecting perceived violations of norms surrounding authorship and the creative process. This shift may also contribute to a form of ontological unease when observers confront the possibility that algorithmic systems can produce artifacts resembling human expression without corresponding intentionality or internal states.

Together, these attributional and affective dynamics may help explain the consistent devaluation of AI-generated outputs reported in prior work (Millet et al., 2023; Ragot et al., 2020; Bellaiche et al., 2023), as well as reduced willingness to attribute authorship or fully recognize such outputs as art (Mikalonytė and Kneer, 2022; Köbis and Mossink, 2021). Rather than reflecting differences in perceptual quality, these responses suggest that AI-generated outputs may be experienced as destabilizing the boundaries of what counts as art.

### 1.1 Tracing the argument: section overview

These expectations and perceived disruptions establish the foundation for the sections that follow. Section 2 examines lay theories of creativity, focusing on intuitions about effort, insight, and inspiration—the “magical” elements audiences attribute to the creative process and infer as embedded in the artifact through the creator’s performance. Prior theoretical frameworks positioned artistic value as emerging from a singular, effortful, and insight-driven human act, while generative AI challenges these assumptions by rendering creative processes more procedural and less visibly tied to human agency and inspiration.

Section 3 situates these intuitions within established psychological frameworks and examines how mechanisms such as agency detection, magical contagion, the extended self, artistic communication, and scarcity jointly structure the experience of artworks as meaningful, intentional, and valuable. Across these mechanisms, artworks are treated not merely as objects, but as traces of human creative performance, indexed by effort, expressive intent, and singularity. This section further examines how AI-generated art may disrupt these interpretive conditions by redistributing perceived agency, reducing cues of embodied effort, and weakening the inference of communicative intent.

Section 4 examines responses to AI-generated art through three organizational framings—tool, collaborator, and threat—illustrating how differences in perceived agency and transparency of generative processes shape observers’ ability to map artifacts onto human creative activity. Whereas the tool and collaborator framings preserve this mapping by maintaining cues of intention, effort, and curatorial control, the threat framing emerges when audiences perceive AI-generated processes as bypassing human creative performance, thereby failing to engage the psychological frameworks that support artistic valuation.

Collectively, Sections 2–4 trace the relationship between creative performance, perceived agency, and evaluative response, establishing the conceptual and psychological groundwork for Section 5, which formalizes hypotheses regarding when and why AI-generated works may be devalued relative to human-created works.

## 2 Lay theories of creativity: intertwined intuitions of “magic” in artistic creation

Beyond formal aesthetic analysis, audience responses to art are profoundly shaped by culturally ingrained lay theories of creativity (Plucker and Runco, 1998). These implicit frameworks structure expectations regarding how creative works should originate and how inferred creative processes contribute to judgments of authenticity (Bullot and Reber, 2013). Cultural narratives surrounding creativity often portray artistic insight as mysterious, ineffable, or inspiration-driven (Simonton, 2018; Weisberg, 2006). This reliance on process-based cues is evidenced by the effort heuristic, in which perceived labor systematically influences judgments of quality (Kruger et al., 2004). Such aesthetic-experiential frameworks extend even to highly technical domains, where intuitive and embodied judgment continues to shape evaluation (Kozbelt et al., 2012). In the context of computational creativity, these findings highlight how judgments of value depend on an inferred history of production rather than surface features alone.

Furnham (1988) proposed that lay theories function as essential, albeit technically incomplete, belief systems that allow individuals to navigate and attribute meaning to complex phenomena. In the domain of aesthetics, these theories prioritize the presence of a ‘human trace’—inferred intention, effort, or authorship—as a central basis for value (Newman and Bloom, 2012), a concern that is also reflected in philosophical accounts of authenticity and artistic origin (Jago, 2019). Absorbed through cultural narratives rather than explicit instruction, these frameworks portray artistic creation as arising from more than technical skill or deliberate effort. Central to this belief structure is the expectation of a creative “spark”—an ineffable quality that researchers associated with inspiration, insight, and authentic expression (Kant, 1790/2000; Kounios and Beeman, 2014; Newman and Bloom, 2012) and the idea of innate genius (Sternberg, 1985). Rather than isolated beliefs, these lay theories operate as an integrated system governing expectations for both creation and reception.

Although creativity is actively being redefined to accommodate AI-generated processes(Wingström et al., 2024), this analysis proposes that evaluations of creative work by lay audiences remain shaped by lay theories of creativity and the psychological mechanisms used to infer human creative performance, which privilege embodied effort, nonlinear insight, and human agency (Kruger et al., 2004; Newman and Smith, 2016). When AI-mediated processes appear to bypass these expected pathways—through the proceduralization of generative activity, curatorial workflows, or the perceived outsourcing of insight generation—such shifts are likely to be interpreted as a violation of lay theories of creativity and authorship, not merely as technical innovation. This perceived disruption may alter how the artifact is mapped onto human agency, shaping appraisals of external causation, reduced controllability, and high replicability. These appraisals, which are consistent with attribution-based models of emotion proposed by Weiner (1985) and Scherer (2001), are theorized to give rise to anger and ontological unease in responses to generative technologies.

### 2.1 The myth of inspiration and the “Aha!” moment

A central element of lay theories of creativity is the “myth of inspiration,” often imagined as a sudden “Aha!” moment in which an external muse bestows artistic insight (Most, 2018; Trivigno, 2020). The term *inspire*, derived from the Latin *inspirare* (“to breathe into”), reflects early mystical conceptions of creativity as a divine gift. During the Romantic era, as theorists reconceptualized this “breath” as an internal faculty, the spark of creation evolved from a divine infusion into a marker of innate genius (Kant, 1790/2000). Consequently, the individual creator became the privileged originating source of the work (Sternberg, 1985; Plucker and Runco, 1998). By relocating this creative impetus to the human psyche, the Romantic framework redefined artistic output as an index of human essence—a belief that continues to underpin modern expectations of authenticity (Gelman, 2003; Newman et al., 2011; Hood, 2009). This legacy helps explain why algorithmic generation is experienced not merely as a technical shift, but as a challenge to foundational assumptions regarding creativity and authorship (Mikalonytė and Kneer, 2022; Millet et al., 2023); in this view, AI acts as an implicit violation of long-standing lay theories.

Cultural fascination with prodigies and solitary creators continues to reinforce this narrative, portraying creativity as a mystical personal essence rather than iterative recombination (Gardner, 1993; Simonton, 1994). Within this framework, the spontaneous breakthrough functions as evidence of that essence. The tale of Archimedes’ “Eureka!” moment remains emblematic of insight appearing to emerge beyond calculation, although what appears magical reflects the mind’s largely unconscious integration and reorganization of information.

According to Wallas’s (1926) influential stage model, the “Aha!” moment (insight/illumination) is preceded by preparation—deliberate, often effortful engagement—and incubation, a period during which information is processed outside conscious awareness. During this incubation phase, information is iteratively reorganized until it becomes available to conscious thought. In formal accounts of this model, the transition is experienced subjectively as the “spark”—the sudden and salient emergence of insight into awareness.

Contemporary cognitive scientists have reframed this account by treating these “stages” not as discrete, sequential phases, but as overlapping and dynamically interacting processes (e.g., Jung-Beeman et al., 2004; Kounios and Beeman, 2014). Simonton (1988, 2018) characterized this as the iterative reorganization of mental representations until a novel configuration emerges. In this view, the “Aha!” moment does not constitute a distinct stage, but instead marks the threshold at which ongoing, distributed processing becomes accessible as sudden clarity. What feels “magical” to the lay observer reflects the restructuring of information through an integration process.

### 2.2 Generative AI: the perceived demystification and externalization of the ‘Aha!’ moment

Generative AI may disrupt the narrative of inspiration by altering how effort and intentionality are mapped onto artifacts. Even when AI-generated works satisfy formal product-based criteria for creativity—novelty, utility, and surprise—which Simonton (2018) characterized as essential criteria, audiences may experience a disconnect between the artifact and the expected human generative process. According to the effort heuristic, perceived labor could function as a proxy for artistic quality (Kruger et al., 2004), automated generation complicates the inference that creative output reflects effortful human cognition.

At the same time, AI systems instantiate generative processes that resemble formal accounts of associative creativity. By producing outputs through large-scale probabilistic recombination, these systems foreground the possibility that creative outcomes arise from structured search across patterns of association in representational space—a principle consistent with associative theories of creativity (Mednick, 1962) and developed in contemporary accounts of combinatorial and search-based models of creative cognition (e.g., Simonton, 2018; Boden, 2004; Wiggins, 2006). While observers may recognize that the human retains curatorial oversight, the AI contributes outputs that cannot be fully anticipated. McCormack et al. (2019) argued that such unpredictability can function as a source of emergent surprise, allowing the collaborator framing to treat this distance between intention and outcome not as a loss of control, but as the site of generative emergence itself. Crucially, however, this generation of autonomous surprise simultaneously demystifies the traditional ‘aha’ moment, effectively outsourcing the ‘magic’ of the creative performance to an algorithmic process.

In this sense, AI does not eliminate the “aha!” moment, but repositions it as an emergent property of distributed, non-local recombinatory processes rather than a discrete event within an embodied human mind.

When algorithmic systems produce outputs that resemble the results of human insight, the inference that such outputs originate from uniquely human processes becomes less stable. Because lay theories of creativity depend on inferences about agency, effort, and intentional transformation, attributions of “magic” are weakened when these generative processes are externally visible or plausibly mechanized. What is experienced as magical is thus reinterpreted as the product of structured generative procedures rather than ineffable insight.

While scholars such as Cropley (2025) argued that AI remains confined to statistical mimicry, its performance suggests that even high-level human creativity—described by Beghetto and Kaufman (2007) as “Pro-c” or “Big-C”—may overlap structurally with algorithmic recombination. When the “Aha!” moment is interpreted through this mechanistic lens, the distinction between insight as subjective experience and insight as a computationally tractable process becomes less stable, a shift consistent with observations by Newman and Bloom (2012). Artistic valuation often depends on the attribution of a human trace—evidence that insight emerged from embodied attention and effort. When generative processes are attributed to computational systems rather than human minds, this intuition is weakened. Ultimately, AI does not remove the “spark,” but shifts its explanatory status from ineffable insight to structured generation.

If insight is not confined to internal generative activity, then its locus may instead be distributed across the interaction between human users and generative systems. In human–AI creative interaction, insight may therefore be less accurately understood as a discrete internal “Aha!” moment and more as an emergent property of distributed cognition. This view aligns with the theory of the extended mind proposed by Clark and Chalmers (1998) and is supported by contemporary research into iterative human–system coupling and reinterpretation (Ashkinaze et al., 2024; Kounios and Beeman, 2014).

### 2.3 The prompter: disembodied creativity and cognitive style shift

Demystification reshapes perceptions of the human prompter. Within the ‘genius/spark’ framework, artistic value has been tied to creative performance—the visible, embodied transformation of an idea into material form (Newman and Smith, 2018; Wessell, 1978). Aesthetic judgment tracks the observable “action” of creation: traces of problem-solving and iterative engagement with a medium (Kozbelt et al., 2012). When AI serves as the primary generator, the prompter’s role shifts from execution to orchestration (McCormack et al., 2019). Casacuberta and Guersenzvaig (2025) characterized this as “disembodied creativity,” in which the creator is partially detached from the material feedback loop. Without direct causal interaction with the medium, the work may fail the Lay Expectation of Evident Effort—the belief that authentic artistic value requires visible struggle, a phenomenon identified in recent empirical studies (Bellaiche et al., 2023; Morris and Alvey, 2024).

AI shifts the locus of creative cognition from associative, divergent recombination to collaborative navigation of latent space—a shift consistent with the exploratory and transformational categories proposed by Boden (2004) and observed by Cetinic and She (2022). Agency is no longer traced in embodied struggle or iterative negotiation with material resistance, but in high-level selection, semantic framing, and evaluative decision-making. While traditional media provided material resistance—the technical and conceptual demands that Dewey (1934) described as requiring creators to negotiate intent with a physical medium—AI interaction replaces this material negotiation with semantic negotiation. Semantic negotiation refers to the meta-cognitive and symbolic effort involved when a prompter navigates the probabilistic constraints of a generative model to achieve a specific intentional outcome. In this framework, the prompter does not bypass cognitive labor; rather, they refine intent through iterative engagement with the model’s outputs—a process characterized by McCormack et al. (2019) and Casacuberta and Guersenzvaig (2025) as a recursive feedback loop. Expert prompting, in this sense, constitutes a specialized, learned form of human skill, shaping coherence and meaning even as the associative work is performed by the model.

The audience’s struggle to recognize human effort in AI art is often reinforced by the ‘Romance of Computation,’ a framework in which Dexter et al. (2011) described the tendency to attribute autonomous creative agency to machines. As Epstein et al. (2020) observed, this narrative often obscures the role of human input by leading observers to overlook the prompter’s generative contributions. Consequently, while the prompter remains the locus of initial generative direction—structuring the iterative prompt–system interaction that produces the artifact—the observer does not attribute the resulting output to a coherent human creative performance. Instead, the prompter is often reclassified as an evaluative curator rather than a generative agent. This shift weakens the perceived link between artifact and human creative agency (Daniele & Song, 2019) and ultimately disrupts the process of agency mapping.

Within expert practice, Wingström et al. (2024) argued that prompting involves nuanced directive and interpretive control over model outputs—a process they characterized as highly specialized—but this form of agency is not readily legible as part of the artifact’s perceived creative origins. Importantly, observers rely on culturally ingrained assumptions that art should embody a trace of human creative performance. In this context, the “effort heuristic” identified by Kruger et al. (2004) functions as a primary cue supporting the attribution of human agency; however, effort alone is not sufficient. When generative processes are perceived as procedurally mediated rather than grounded in human action, the artifact is less readily imbued with the properties of authorship and intentionality that underpin lay theories of creativity.

In sum, AI art prompts a renegotiation of lay theories along multiple dimensions: it relocates the moment of insight from the spontaneous “spark” to iterative curation, reconfigures the embodied linkage between artist and artifact, and transforms cognitive labor into meta-cognitive, strategic orchestration. These shifts are emotionally charged: the move from effortful “struggle” to high-level “semantic navigation” results in negative appraisals, including ontological unease and aesthetic anger, reflecting friction between human-centric definitions of art and emerging collaborative paradigms.

## 3 Agency mapping and the psychological foundations of artistic value

The human-centric schema of art—rooted in Romantic-era conceptions of creativity—assumes that artworks are the expression of a singular human creative performance with a traceable generative source. Audiences use this human-centric schema to inform a coordinated set of psychological mechanisms through which observers infer meaning, intention, and value. These mechanisms—including the concept of the extended self (Belk, 1988), agency detection (Epley et al., 2007), the artistic design stance (Bullot and Reber, 2013), and the role of magical contagion (Newman and Bloom, 2012)—function together as an agency mapping system. This system, which also incorporates principles of artistic communication (Dolese et al., 2014) and perceived scarcity (Kruger et al., 2004), anchors the artwork to a perceived human source. When AI disrupts this system, it produces predictable emotional consequences: when observers cannot reconstruct a human generative pathway, they often experience “aesthetic anger” at the violation of creative norms and “ontological unease” regarding creations unanchored in human cognition. These responses arise not from superficial dislike, but from the perceived breakdown of mechanisms that normally anchor meaning and social grounding to human authorship.

Artistic value, in this sense, is not derived from the artifact alone but from the observer’s ability to map it onto a plausible history of human cognition and embodied performance. When this mapping succeeds, the work is experienced as an expression of a mind; when it fails, the artifact risks appearing procedural, ungrounded, or socially inert. AI-generated art is uniquely positioned to disrupt this system because it obscures or replaces the cues that these mechanisms rely on to anchor human agency: the locus of authorship shifts away from a singular human source, the human mind behind the generative process becomes displaced, and effort is no longer reconstructible. Consequently, attribution processes are strained across multiple cognitive levels simultaneously.

This section examines how each mechanism contributes to agency mapping and why its disruption leads to systematic devaluation, resistance, anger, and ontological unease. Rather than reflecting a single deficit, negative responses to AI-generated art emerge from a cumulative failure to sustain the perception of a human-originated creative act. Importantly, these mechanisms operate on the presupposition of not just agency, but specifically human agency—an assumption that becomes unstable under AI-mediated production.

### 3.1 Scarcity as an effort heuristic

Artistic value is related to scarcity in the sense that a work is implicitly believed to be the singular outcome of a unique creative act. Benjamin (1936/1968) proposed the concept of the “aura”—the perceived authenticity and unique presence of a work in time and space—to highlight how originality and uniqueness anchor perceptions of authenticity (Gelan, 2020). Psychologically, audiences rely on scarcity as an effort heuristic (Kruger et al., 2004): rare works signal that the artist’s performance was singular, irreplaceable, and required substantial effort (Newman and Bloom, 2012; Bellaiche et al., 2023). AI art undermines this heuristic through its capacity to generate countless high-fidelity variations with negligible friction. Consequently, the “aura” collapses, a shift that destabilizes traditional valuation systems and weakens the observer’s ability to reconstruct human effort, thereby reinforcing the perception that AI art lacks intrinsic worth (Magni et al., 2024; Morris and Alvey, 2024).

### 3.2 The extended self: tools as conduits of agency

The perceived erosion of the aura reflects a disruption of the “extended self.” Belk (1988, 1989) proposed that material objects can become incorporated into a person’s identity, functioning as symbolic extensions of the self. Building on this framework, Newman and Smith (2018) argued that artworks serve as paradigmatic extended-self objects, insofar as they are interpreted as repositories of a creator’s identity, effort, and embodied performance.

In artistic contexts, this sense of self-extension is supported by the perceived connection between the artist and their tools. As Newman and Bloom (2012) argued, the value of an artwork is closely tied to the physical process of its creation; instruments such as brushes and chisels serve as conduits through which human intention is manifested in material form. When the creative process is perceived as transparent and the tool as continuous with bodily action, observers are more readily able to interpret the resulting artifact as a material continuation of the creator’s embodied agency (Belk, 1988, 1989; Newman and Smith, 2018).

AI systems, however, are less often experienced as transparent tools. Instead, observers frequently perceive them as semi-autonomous systems operating according to opaque generative processes (Epstein et al., 2020). Unlike traditional artistic instruments, which recede during skilled use, AI systems tend to remain experientially salient as independent sources of generative output. This perceived opacity is associated with attributions of partial autonomy or system-level agency, which in turn can decouple the human creator from the final artifact (Magni et al., 2024).

Consistent with this shift in perceived agency structure, observers are more likely to attribute reduced intentionality and creativity to AI-generated works relative to human-made ones (Chamberlain et al., 2018; Bellaiche et al., 2023; de Rooij, 2025; Magni et al., 2024). Importantly, this effect persists even when human involvement is present, suggesting that what changes is not the actual causal role of the human, but the perceptual accessibility of that role within the artifact. As a result, AI-generated outputs are less likely to be experienced as direct extensions of the creator’s self, even when they originate within human-guided workflows.

### 3.3 The artistic design stance and agency detection: expecting intention behind art

Bullot and Reber (2013) argued that audiences adopt an artistic design stance—a default orientation toward artworks as intentional acts. Viewers interpret brushstrokes, composition, and style as deliberate choices that express meaning, an interpretive framework that presupposes a conscious agent (Bullot and Reber, 2013; Dolese et al., 2014; Takahashi, 1995). Humans are also predisposed to perceive intention in meaningful patterns through an agency-detection bias (Heider and Simmel, 1944; Guthrie, 1995; Epley et al., 2007), which inclines audiences to assume that artworks originate from conscious, purposeful agents. Together, these mechanisms allow viewers to experience artistic features as traces of intentional human activity embedded within the artifact itself (Newman and Smith, 2018).

AI-generated art disrupts both of these cognitive tendencies. Although audiences may appreciate the surface aesthetics of AI-generated works, the perceived absence of a subjective mind or lived experience can make it more difficult to attribute authentic intention to the artifact. For example, Mikalonytė and Kneer (2022) found that participants judged robot-produced paintings as art to a similar extent as human-produced ones, yet were significantly less willing to consider the robots themselves “artists,” reflecting reduced attributions of artistic intention. Terzidis et al. (2023, p. 1715) described this phenomenon as “unintentional intentionality,” in which a machine produces the appearance of purposeful design without an underlying conscious source. In this sense, AI-generated works may partially satisfy the perceptual features associated with the artistic design stance while weakening the inference that those features originate from a situated human subject.

### 3.4 Magical contagion: the human touch as creative essence

Magical contagion—the belief that physical contact transfers an invisible essence from person to object (Frazer, 1890/1959; Mauss, 2005; Rozin and Neneroff, 2002)—operates as a psychological mechanism that links the artist’s embodied performance, intentionality, and the aura of the extended self to the artifact. Newman and Bloom (2012) demonstrated that the value of an original artwork lies not in perceptual features alone, but in the belief that the artist’s physical presence confers authenticity. Within this framework, perceived effort functions as a diagnostic cue: the “effort heuristic” (Kruger et al., 2004) allows observers to infer the depth of creative essence transferred, connecting the human mind, body, and intentional action to the artifact itself. When this chain is broken—as in AI-generated art, the artifact loses the aura of the extended self and the perceivable trace of human intention, undermining its perceived authenticity and value.

This process is further mediated by sensitivity to “human marks”—idiosyncratic traces of manual execution conveying motor origins (Takahashi, 1995; Chamberlain et al., 2018). Chamberlain et al. (2018) found that perceptual features tied to natural human movement—such as the dynamics and timing of strokes—enhance aesthetic engagement. Importantly, these human marks serve not merely as signs of labor, but as cues that an originating insight occurred; through these traces, observers perceive authorship as transferring to the artifact (Newman and Bloom, 2012). AI-generated art disrupts this transmission. Although a human may provide prompts, a non-biological system executes the transformation, disrupting the extended self-mechanism. Without effort cues or subtle motor signatures, the conduit between creator and artifact attenuates, and the human prompter’s agency becomes difficult for observers to register, weakening the symbolic link required for inter-agentic communication.

### 3.5 Artistic communication: art as a social dynamic

Art is not only valued as intentional but also as communicative in a qualified sense. Dolese et al. (2014) and Dolese (2025) did not posit a direct transmission of meaning from artist to viewer. Rather, they conceived of artworks as containers of embodied constraints that structure interpretation, allowing viewers to *infer* the presence of an originating perspective. In this sense, the artwork functions as a bridge—not between fixed meanings, but between a creator’s situated engagement with experience and the viewer’s reconstructive, interpretive activity.

Appreciation is enhanced when viewers can construct a narrative or perceived “aboutness” behind a work, an inference more readily anchored to human creators (Bellaiche et al., 2023). Fundamentally, this process depends on the assumption that the artifact is the product of a mind engaged in the world. When this assumption weakens—as in AI-generated art, where the generative pathway bypasses or obscures a traceable human source (Section 2)—the viewer’s ability to sustain this communicative inference is disrupted. Rather than failing to decode a message, the viewer struggles to justify the presence of a communicative act at all, and the work may be experienced as a socially sterile or indeterminate simulation.

### 3.6 Synthesis: the structural disruption of agency mapping

Across these mechanisms, a unifying functional requirement emerges: audiences evaluate artworks by tracing a perceived pathway from artifact to human generative source. Observers rely on cues such as scarcity (perceived uniqueness), embodied continuity (extended self), contact-based associations (magical contagion), inferred stylistic choices (artistic design stance), and communicative constraints (artistic communication) to anchor a work in human agency. These mechanisms are interdependent: the perception of a rare, effortful act reinforces the sense of embodied involvement; assumptions of physical or causal connection signal continued authorial engagement; and communicative interpretation presupposes that such traces reflect intentionality and situated experience.

Artistic value, therefore, emerges not from the artifact’s surface features alone, but from the observer’s ability to reconstruct a plausible history of human cognition and embodied performance. When this mapping succeeds, the work is experienced as expressive, socially grounded, and animated by human intentionality. When it fails, the artifact risks appearing procedural, unanchored, or socially inert, as though it emerged without meaningful human involvement.

AI-mediated production disrupts this mapping process by displacing the perceived locus of generative labor. The creative process shifts from tangible negotiation with a medium to semiotic negotiation with a probabilistic system—a form of labor that remains largely invisible to observers. Unlike photography or digital painting, where the observer typically perceives the tool as a passive vessel for human agency, AI systems are often experienced as participating directly in the generative process itself. As a result, the final artifact becomes psychologically decoupled from the prompter’s embodied performance, leaving the human role perceived as managerial, curatorial, or supervisory rather than fully creative.

The emergent consequence is a structural breakdown in agency mapping. Observers encounter works that are formally coherent yet difficult to anchor to a human generative source. This disruption produces characteristic emotional responses: aesthetic anger, reflecting frustration at violations of expected agency attribution and human-centered creative norms, and ontological unease—the existential friction of confronting outputs that appear meaningfully structured despite lacking a clearly reconstructible human origin. These responses reveal how AI-mediated production destabilizes the perceptual and interpretive scaffolding that ordinarily grounds art in embodied human activity.

This systemic ambiguity necessitates a shift in how observers interpret the artifact. As the direct link to a human source becomes obscured, audiences move between different interpretive strategies to resolve the uncertainty introduced by AI-mediated production.

## 4 Organizing frames, structural violations, and emotional responses in AI art

As established in Section 3, artistic valuation depends on the observer’s ability to map an artifact onto a reconstructible history of human generative activity. When this mapping becomes uncertain or breaks down, observers do not respond uniformly. Instead, they adopt interpretive framings that attempt to stabilize or account for the disrupted relationship between artifact and human agency.

Discourse around AI-generated art organizes into three dominant framings: AI as tool, AI as collaborator, and AI as threat. These framings are not merely descriptive; they reflect different ways of interpreting how AI-mediated processes engage—or fail to engage—the lay theories and psychological mechanisms that underpin artistic value. Through tool and collaborator framings, observers preserve or redistribute their perception of human creative involvement, allowing observers to maintain partial alignment with expectations of effort, agency, intention, contagion, and communicative intent. However, when the perception of these cues is sufficiently bypassed—when effort flattens, agency redistributes, and the artifact is experienced primarily as the product of a procedural system rather than a human generative path—the mapping collapses and the work is more likely to be interpreted through a threat framing.

The emotional responses associated with AI-generated art follow from these interpretive outcomes. Aesthetic anger emerges when the observer appraises the breakdown as a violation of the norms governing human creativity—specifically intention, effort, authorship, or creative performance. Ontological unease arises when the breakdown destabilizes the category itself, calling into question the fundamental assumption that creative artifacts are expressions of human minds.

The following sections examine how each framing organizes perception under conditions of disrupted agency mapping, and how the degree to which an observer can infer human generative activity predicts both valuation and emotional response.

### 4.1 AI as tool: preserving human agency through instruments

In the tool framing, AI is interpreted as an extension of human agency, analogous to traditional creative instruments such as brushes, chisels, or cameras. This framing relies on the extended self-hypothesis; in their respective formulations, Belk (1988, 1989) and Newman and Smith (2018) posited that tools can function as transparent conduits of embodied human intention and creative performance. When observers perceive the technology as a passive instrument, the human prompter retains authorship, and the AI output is experienced as a material instantiation of human insight rather than the product of a semi-autonomous system. For this framing to remain stable, the audience must successfully employ the artistic design stance (Bullot and Reber, 2013), decoding the human’s “why” behind each formal choice to recognize intentionality and expressive meaning.

Human valuation of creative products is deeply tied to magical contagion, the belief that physical contact or proximity transfers an enduring essence from person to object (Frazer, 1890/1959; Rozin and Neneroff, 2002; Mauss, 2005). In traditional artistic practice, instruments function as vessels for this contagion: brushes, pens, and chisels channel the artist’s internal “spark” into material form, leaving behind perceptible human marks—subtle traces of motor execution, dynamics, and timing of strokes—that audiences interpret as evidence of effort and creative insight. This externalization is reinforced by the effort heuristic, whereby observers use visible struggle and time investment as proxies for the depth of the creative essence transferred (Kruger et al., 2004; Newman and Bloom, 2012). Chamberlain et al. (2018) demonstrated that audiences respond positively to perceptual cues of human motor activity—such as brushstroke dynamics or idiosyncratic marks—that signal an embodied, human origin. Furthermore, the knowledge that an artist labored extensively on a piece (“time presence”) enhanced its perceived authenticity and value (Morris and Alvey, 2024; Bellaiche et al., 2023; Magni et al., 2024). In these cases, AI can be perceived as a transparent extension of human agency only if such cues remain legible and attributable to the human prompter.

Historically, similar legitimacy challenges arose with new technologies. When photography emerged, it was initially dismissed as a mechanical process lacking the “soul” of manual execution (Baudelaire, 1980). The medium gained acceptance as a creative tool once audiences learned to map photographs back to the photographer’s embodied presence (Maynard, 1997); framing, timing, and perspective became perceptible signatures of human intention, preserving the pathway for magical contagion. Likewise, a tool framing for AI requires that the observer recognize the human’s curatorial choices—the selection of prompts, guidance of outputs, and iterative refinement—as the locus of creativity.

AI disrupts the traditional boundary between creator and instrument, as the system’s high-level execution often appears to mimic the autonomous insight previously associated with the human mind. While the human prompter provides inspiration or constraints, the visible transformation occurs within an algorithmic system. Because the resulting artifact often lacks the idiosyncratic motor traces and visible effort cues that convey embodied agency, audiences may struggle to perceive the human “spark” as materially inscribed. This failure weakens the extension of the self (Belk, 1988, 1989) and the symbolic and affective transmission that sustains inter-agentic communication. A perceptual gap is supported by previous research: audiences attributed less creativity and intentionality to AI-generated works than to human-made ones (Chamberlain et al., 2018; Bellaiche et al., 2023; Magni et al., 2024; de Rooij, 2025), even when humans were involved in curatorial or prompt-guiding roles.

Furthermore, AI’s procedural and semi-autonomous operation threatens the magical contagion pathway. Without perceivable human effort or struggle, observers default to attributing agency to the algorithm rather than to the human. Even if surface aesthetics are compelling, the work appears procedural rather than expressive; the human signature becomes illegible, and the artifact risks being interpreted as socially and emotionally detached. Maintaining a tool framing thus depends on strategies that preserve human legibility: emphasizing deliberate choices, iterative refinement, and curatorial oversight allows audiences to anchor agency and creative insight in the human, rather than the AI.

In sum, interpreting AI as a tool requires preserving or signaling the cues that link human intention, effort, and embodied execution to the final artifact. By maintaining these markers, observers can experience the output as an extension of the creator’s self, sustaining magical contagion, the extended self, and the artistic design stance. Failure to preserve these cues may collapse the tool framing, pushing audiences toward collaborator or threat interpretations, where the AI is perceived as autonomous, human intention becomes detached from the artifact, and perceived structural violations of creative expectations elicit aesthetic anger, ontological unease, or devaluation of the work.

### 4.2 AI as collaborator: distributed agency

Beyond the tool framing, the collaborator framing positions the algorithm not as a passive instrument but as a co-creative partner, with humans and machines jointly shaping the final artifact (Epstein et al., 2020). Historically, collaboration with non-human generative systems—such as electronic synthesizers or algorithmic composition systems—was theorized as challenging traditional boundaries of creative agency (Boden, 2004). Within this partnership, AI functions as a generative medium where, as Akten (2021) posited, operation occurs not through direct manual control, but through complex, data-driven transformations within a high-dimensional space where the mapping from human intention to final output is inherently non-linear and non-transparent.

Empirical research suggested that humans navigate this collaborative space through distinct interaction styles, often acting as high-level directors who shape, evaluate, and integrate machine outputs rather than specifying every detail of the generative process (Ostern et al., 2024). In these settings, the human role shifts toward curating and validating outputs to ensure alignment with intended goals. Even when the system appears partially autonomous, the human remains the primary locus of intentional direction, accountability, and evaluative constraint (Akten, 2021; Epstein et al., 2020). Consequently, agency is experienced as distributed: the human provides vision, selection, and interpretive grounding, while the system contributes stochastic variation and emergent novelty.

However, this attribution of shared agency remains fragile. Köbis and Mossink (2021) demonstrated that even when observers cannot reliably distinguish between human- and AI-generated outputs, revealing the machine’s role nevertheless produces systematic, source-based devaluation of the work. Observers may acknowledge the novelty or aesthetic richness of AI-assisted outputs while simultaneously questioning whether the human retains sufficient authorship to satisfy the artistic design stance. Because the mapping from human intention to final outcome remains partially opaque within AI-mediated systems (Akten, 2021), the source of novelty is no longer attributed solely to the human creator.

For some observers, this redistribution of generative agency produces a form of evaluative tension or ontological unease. The work may remain aesthetically compelling while simultaneously signaling the loss of a unique and unified human signature. Because AI systems are often perceived as lacking the “internal motivation” or “subjective experience” associated with authentic expression (Castelo et al., 2019), the collaboration is experienced as fundamentally asymmetrical. The audience can appreciate the artifact aesthetically while struggling to reconstruct a coherent human generative history behind it.

This tension becomes especially pronounced when artworks are evaluated as communicative acts rather than merely formal objects. Art is valued not solely for aesthetic form, but as a bridge between the creator’s lived experience and the viewer’s interpretive world (Dolese et al., 2014; Dolese, 2025). When a generative AI contributes substantively to the production process, this communicative bridge weakens because the system is perceived as unable to embody or transmit subjective experience. Even when a human prompter provides substantial curatorial guidance, aspects of the artifact may appear unanchored from a coherent lived perspective. The audience may continue to enjoy the work aesthetically, yet the social and epistemic link to a conscious human agent—the foundation of shared artistic communication—becomes destabilized.

Consequently, the extended self relationship between creator and artifact also weakens, as the boundary between human and machine agency becomes increasingly blurred. The artifact risks being experienced less as a communicative expression of human subjectivity and more as a simulation of expressive form, signaling human insight only partially or inconsistently. This produces a sense of distributed or plural authorship that is difficult to anchor in a single intentional source, generating epistemic ambiguity in agency attribution. At this stage, responses may shift toward interpretive instability that can, under certain appraisals, transition into threat-based evaluations, generating both aesthetic interest and subtle discomfort.

### 4.3 AI as threat: the synthesis of structural disruptions

The threat framing does not emerge from a single violated expectation, but from the convergence of disruptions across the psychological and cultural systems that jointly sustain artistic value. When effort, agency, insight, intentionality, communicative grounding, and scarcity cues are simultaneously destabilized, the artifact can no longer be mapped onto a coherent model of human creative production. This cumulative breakdown—rather than any individual violation—gives rise to aesthetic anger and ontological unease.

Creative artifacts are culturally understood not only as products, but as outcomes of a specific kind of human generative process, characterized by embodied effort, intentional transformation, and situated cognitive engagement. Observers therefore do not evaluate artworks solely by their formal properties; they evaluate them by reconstructing the inferred human pathway through which those properties were produced. When AI-mediated generation disrupts the availability or coherence of these process cues, the causal link between artifact and human creative activity becomes difficult to sustain.

This disruption produces a shift in evaluative inference: the artifact may remain formally intelligible as an artwork, yet the underlying generative process becomes ambiguous. Here, the distinction between type authenticity and token authenticity (Jago, 2019) becomes important. AI systems often preserve type authenticity, in that they reproduce the recognizable stylistic and structural features of artistic categories. However, they may weaken token authenticity by obscuring the specific human cognitive and historical pathway through which the artifact was generated. Importantly, this is not a failure of appearance, but a failure of process traceability.

The resulting instability is best understood as a breakdown in process-based grounding of category membership. Within lay theories of creativity (Section 2), “art” is partially defined by the presumed presence of effortful, intentional, and embodied human cognition. Within the agency mapping system (Section 3), these same assumptions function as inferential cues that anchor meaning and authorship to a human source. When token authenticity cannot be reliably reconstructed, these cues no longer integrate into a coherent attribution of human creative agency.

In this sense, the threat framing is not produced by perceptual novelty or stylistic excess, but by a structural incongruity between preserved category features (type authenticity) and disrupted process attribution (token authenticity). When observers cannot reconcile these dimensions, the artifact becomes difficult to situate within a stable model of human-originated creation. The result is not merely reduced valuation, but a weakening of the conditions under which the artifact is confidently categorized as the product of human artistic activity.

Aesthetic anger follows from violations of expected human effort and intentional authorship; ontological unease emerges when the artifact cannot be securely anchored within a human generative framework that stabilizes its meaning as art. Importantly, this response is not uniform across observers but varies with the degree to which process-based cues are required for category membership inference.

In this sense, the threat framing is not produced by formal failure, but by the structural incongruity between high type authenticity and weakened token authenticity. When observers can no longer reconcile these dimensions, effort, agency, insight, intentionality, communicative grounding, and scarcity cues fail to cohere into a unified model of human creative production. The resulting percept is not simply that the artifact is “AI-generated,” but that the underlying structure of creative causation has become opaque, decentered, and psychologically unmappable. It is this collapse of coherent attribution—rather than any single violated expectation—that gives rise to aesthetic anger and ontological unease.

### 4.4 Integration: the structure of the perceptual gap

Across the three framings, audience responses are organized by a common underlying process: the extent to which observers can reconstruct a coherent history of human generative agency from the artifact. Rather than responding directly to surface features of AI-mediated works, observers attempt to infer the causal chain linking human intention, action, and expressive outcome. The stability of this inferential reconstruction determines whether AI is interpreted as a tool, collaborator, or threat.

Within the tool framing, this reconstruction remains relatively transparent: effort, intention, and expressive control are successfully mapped onto the human prompter, allowing the artifact to be experienced as an extension of embodied human agency (Belk, 1988, 1989; Kruger et al., 2004). In the collaborator framing, this mapping becomes partial but still recoverable, as agency is distributed across human selection, guidance, and machine generation (Epstein et al., 2020; Akten, 2021). Here, observers tolerate uncertainty by treating opacity as a site of emergent novelty rather than loss of authorship (McCormack et al., 2019). In the threat framing, however, this reconstruction fails: the causal link between human input and expressive outcome becomes too opaque to stabilize, and the artifact can no longer be reliably anchored in a coherent human generative history.

This variability is closely tied to differences in how observers interpret opacity in generative systems. Wingström et al. (2024) showed that trained practitioners were more likely to interpret AI systems as instruments for extended or distributed expression, whereas lay audiences more often experienced the same systems as epistemically opaque. This difference aligns with Akten’s (2021) account of meaningful control in high-dimensional generative systems: what appears as loss of agency from a lay perspective may still be experienced as structured, intentional interaction from a practitioner’s standpoint. As a result, practitioners are more likely to sustain attribution of agency through iterative selection and interpretive framing, whereas lay observers more readily experience breakdowns in attribution when causal pathways become difficult to reconstruct.

Importantly, these differences do not reflect disagreement about the artifact itself, but rather variation in the perceptual threshold for maintaining a coherent mapping between human lived experience and artistic output. When this mapping is preserved, AI-generated works can be interpreted as continuous with human expressive intention; when it breaks down, the same works are reclassified as procedurally generated outputs lacking stable human grounding. Thus, the transition between tool, collaborator, and threat framings reflects not only differences in technological structure, but differences in the observer’s capacity to sustain agency attribution under conditions of increasing generative opacity.

## 5 Empirical implications and theoretical predictions

The disruption of agency mapping described in previous sections generates a series of empirical predictions regarding how AI-mediated art is evaluated. If artistic value depends on the reconstruction of a coherent human generative history, then variation in the visibility, attribution, and interpretability of human creative cues should systematically influence emotional response, agency attribution, and evaluative judgments.

*Hypothesis 1*: Emotional responses to violations of lay theories of creativity.

AI-generated artworks are expected to evoke distinct emotional responses when they violate observers’ lay theories of creativity. Creative artifacts are culturally expected to arise from human effort, intentionality, and embodied insight. When an artifact is identified as the product of a procedural system rather than a human creator, observers are hypothesized to experience aesthetic anger due to perceived violations of these norms. At the same time, ontological unease is expected to emerge as the boundaries of creative authorship and artistic authenticity become destabilized. Future research could examine whether AI-labeled works reliably elicit stronger anger and existential discomfort than identical artifacts attributed to human creators.

*Hypothesis 2*: Disruption of agency attribution in AI-generated art.

Observers’ valuation of art is closely tied to the attribution of human agency. When an artifact cannot be easily mapped onto a coherent model of human generative activity, perceptions of authenticity, intention, and meaning are expected to weaken. AI-generated works are therefore hypothesized to be judged as less creative, less authentic, less emotionally engaging and less communicative than identical works attributed to human creators. These effects are expected to be mediated by reductions in perceived intentionality and the inferred connection to a human communicative source.

*Hypothesis 3*: Interpretive framing of AI as tool, collaborator, or autonomous generator.

The psychological response to AI-generated art may depend on how the role of AI is framed within the creative process. When AI is presented as a tool that extends human creativity under transparent guidance, observers may preserve attribution of authorship and perceive continuity of human agency. Collaborative framings, in which creative responsibility is shared between human and machine, may produce intermediate responses. In contrast, framings that emphasize AI autonomy and displacement of human effort may intensify perceptions of threat, leading to stronger aesthetic anger and ontological unease. Future research could experimentally manipulate these framings to examine their effects on perceived authenticity, authorship, and emotional engagement.

*Hypothesis 4*: Moderating role of observer expertise.

Individual differences in artistic or technical expertise may moderate responses to AI-generated art. Observers with greater domain knowledge may be more capable of identifying the procedural and curatorial skill involved in prompting, iteration, and selection within AI-mediated creation. As a result, they may be more likely to attribute partial agency to the human creator and may exhibit reduced devaluation of AI-assisted works. Comparative studies could test whether expertise moderates judgments of creativity, authenticity, and intentionality when evaluating AI-generated artifacts.

*Hypothesis 5*: The psychological experience of agency and creative flow.

Beyond observer perception, prompting may also alter the creator’s own experience of agency and causal authorship in the creative process. Traditional artistic production is characterized by a “material dialogue,” a continuous loop of physical action and sensory feedback that supports embodied agency and creative flow. In contrast, AI-mediated creation involves semantic negotiation, where causal influence is exerted through language rather than direct embodied interaction with the medium.

Future research should investigate how this shift affects the creator’s subjective experience of being the primary source of creative outcomes. Although AI-mediated processes may produce high levels of output satisfaction, they may also yield a diminished sense of personal agency and a more fragmented experience of creative flow compared to traditional embodied artistic practices.

## 6 Conclusion: integrating psychological, cultural, and processual accounts of agency in AI art

AI-generated art challenges the psychological and cultural frameworks through which creativity and its products are evaluated. Across the analyses presented in this paper, responses to AI-mediated works are shaped less by surface aesthetic qualities than by how observers reconstruct underlying lay theories of authorship, intentionality, and embodied effort. When artworks are evaluated as the outcome of human intention and historically grounded processes of creation, AI outputs disrupt the cues used to infer these properties, complicating judgments of authenticity and meaning.

The hypotheses advanced in this paper specify the mechanisms through which these disruptions operate. Across conditions, ontological unease and related emotional responses emerge when observers perceive a decoupling of effort, intention, and embodied performance from the artifact. These responses index violations in the inferential systems that ordinarily link visible cues of production to perceived creative agency. Variables such as perceived transparency of effort, framing of human–AI interaction, and domain expertise jointly shape the stability of these attributions.

Within this framework, public and scholarly discourse can be understood as organizing AI under three interpretive framings: tool, collaborator, and threat. As a tool, AI is interpreted as an extension of human agency that preserves continuity with embodied creative control (Belk, 1988, 1989; Newman and Smith, 2016). As a collaborator, AI introduces distributed generative dynamics in which human and system jointly contribute to outcomes that neither could fully anticipate (Epstein et al., 2020). As a threat, however, AI is interpreted as displacing or obscuring the human generative process, undermining assumptions that creativity arises from effortful, intentional, and embodied action. This latter framing is associated with aesthetic anger and ontological unease, particularly when observers cannot reconstruct a coherent human source of creative agency (Magni et al., 2024). For practitioners, however, engagement with AI may involve a cognitive restructuring of creative control, inducing shifts in reflective practice and perceived authorship (Kosmyna et al., 2025; Faiella et al., 2025; DACS, 2024).

Across domains, these effects generalize beyond the visual arts. In music, literature, and educational production, evaluative and emotional responses similarly depend on whether observers can infer a coherent human generative process from available cues such as effort, intentional direction, and experiential grounding. When these cues are attenuated or redistributed, outputs may be experienced as technically proficient yet affectively and epistemically diminished, reflecting weakened access to perceived human agency rather than deficits in formal quality.

Importantly, these interpretive processes are culturally and generationally variable. Younger audiences, including Generation Z, often adopt more integrative evaluative frameworks in which algorithmic generation is treated as compatible with creativity rather than fundamentally opposed to it (Park and McGee, 2023; Lulla et al., 2024). Nevertheless, even within these populations, perceived human involvement—expressed through intention, effort, and communicative engagement—remains a central determinant of perceived artistic value.

Ultimately, AI-generated art does not merely introduce a new class of aesthetic objects; it exposes the inferential structure through which observers attribute creative agency to artifacts. Across tool, collaborator, and threat framings, the critical variable remains the observer’s ability to reconstruct a coherent human generative pathway from available cues. Resistance to AI art, therefore, reflects not simply a preference for traditional media, but a systemic disruption in the cognitive and cultural mechanisms that link observable features of production to inferred human intention and meaning. The emotional responses associated with these disruptions—including aesthetic anger and ontological unease—underscore that artistic value is fundamentally tied to the reconstructability of a coherent human generative pathway within the artifact.

## References

[ref1] Akten M. (2021). Deep Visual Instruments: Realtime Continuous, Meaningful human Control Over Deep Neural Networks for Creative Expression (Doctoral Dissertation). London, United Kingdom: Goldsmiths, University of London.

[ref2] Anantrasirichai N. Bull D. (2022). Artificial intelligence in the creative industries: a review. Artif. Intell. Rev. 55, 589–656. doi: 10.1007/s10462-021-10039-7 PMC12858611 41625196

[ref4] Ashkinaze J. Mendelsohn J. Qiwei L. Budak C. Gilbert E. (2024). How AI ideas affect the creativity, diversity, and evolution of human ideas: evidence from a large, dynamic experiment. Available online at: 10.48550/arXiv.2401.13481 (Accessed May 21, 2026).

[ref5] Baudelaire C. (1980). “The modern public and photography,” in Classic Essays on Photography, (New Haven, CT: Leete’s Island Books), 83–89.

[ref6] Beghetto R. A. Kaufman J. C. (2007). Toward a broader conception of creativity: a case for "mini-c" creativity. Psychol. Aesthet. Creat. Arts 1, 73–79. doi: 10.1037/1931-3896.1.2.73

[ref7] Belk R. W. (1988). Possessions and the extended self. J. Consum. Res. 15, 139–168. doi: 10.1086/209154

[ref8] Belk R. W. (1989). Extended self and extending paradigmatic perspective. J. Consum. Res. 16, 129–132. doi: 10.1086/209202

[ref9] Bellaiche L. Shahi R. Turpin M. H. Ragnhildstveit A. Sprockett S. Barr N. . (2023). Humans versus AI: whether and why we prefer human-created compared to AI-created artwork. Cogn. Res. Princ. Implic. 8:42. doi: 10.1186/s41235-023-00499-6, 37401999 PMC10319694

[ref6005] Benjamin W. (2006). The work of art in the age of mechanical reproduction. In J. Morra & M. Smith (Eds.), Visual Culture: Experiences in Visual Culture (Vol. 4, pp. 114–137). Routledge. London, United Kingdom. (Original work published 1936).

[ref10] Boden M. A. (2004). The Creative Mind: Myths and Mechanisms. London, United Kingdom: Routledge.

[ref11] Bullot N. J. Reber R. (2013). The artful mind meets art history: toward a psycho-historical framework for the science of art appreciation. Behav. Brain Sci. 36, 123–137. doi: 10.1017/S0140525X12000489, 23507091

[ref12] Casacuberta D. Guersenzvaig A. (2025). Disembodied creativity in generative AI: prima facie challenges and limitations of prompting in creative practice. Front. Artif. Intell. 8:1651354. doi: 10.3389/frai.2025.1651354, 40895592 PMC12391906

[ref13] Castelo N. Bos M. W. Lehmann D. R. (2019). Task-dependent algorithm aversion. J. Mark. Res. 56, 809–825. doi: 10.1177/0022243719851788

[ref14] Cetinic E. She J. (2022). Understanding and creating art with AI: review and outlook. ACM Trans. Multimed. Comput. Commun. Appl. (TOMM) 18, 1–22. doi: 10.1145/3475799

[ref15] Chamberlain R. Mullin C. Scheerlinck B. Wagemans J. (2018). Putting the art in artificial: aesthetic responses to computer-generated art. Psychol. Aesthet. Creat. Arts 12, 177–192. doi: 10.1037/aca0000136

[ref16] Clark A. Chalmers D. (1998). The extended mind. Analysis 58, 7–19. doi: 10.1093/analys/58.1.7

[ref17] Crawford K. Paglen T. (2021). Excavating AI: the politics of images in machine learning training sets. AI Soc. 36, 1399–1116. doi: 10.1007/s00146-021-01301-1, 30311153

[ref18] Cropley A. (2025). Defining authentically human creativity in the age of AI [Preprint]. PsyArXiv. doi: 10.31234/osf.io/md74y_v1

[ref19] DACS (2024). Artificial Intelligence and Artists' Work: A Survey of Artists on AI. London, United Kingdom: Design and Artists Copyright Society.

[ref20] Daniele A. Song Y. Z. (2019). AI+ art=human, in Proceedings of the 2019 AAAI/ACM Conference on AI, Ethics, and Society (pp. 155–161).

[ref21] de Rooij A. (2025). Bias against artificial intelligence in visual art: a meta-analysis. Psychology of aesthetics, creativity, and the arts. doi: 10.1037/aca0000833

[ref22] Dewey J. (1934). Art as Experience. Balch: Minton.

[ref23] Dexter S. Dolese M. Seidel A. Kozbelt A. (2011). On the embodied aesthetics of code. Culture Mach. 12, 1–23.

[ref24] Dolese M. J. (2025). Art as a meaningful environment: an integrative model of aesthetic engagement and interpretation. Front. Psychol. 16:1677529. doi: 10.3389/fpsyg.2025.1677529, 41646911 PMC12867917

[ref25] Dolese M. J. Kozbelt A. Hardin C. (2014). Art as communication: employing Gricean principles of communication as a model for art appreciation. Int. J. Image 4, 63–70. doi: 10.18848/2154-8560/cgp/v04i03/44133

[ref9001] Donahue C. McAuley J. Puckette M. (2018). Adversarial audio synthesis. arXiv preprint arXiv:1802.04208.

[ref26] Elgammal A. Liu B. Elhoseiny M. Mazzone M. (2017). CAN: creative adversarial networks, generating" art" by learning about styles and deviating from style norms.

[ref9002] Engel J Agrawal KK Chen S Gulrajani I Donahue C Roberts A. (2019) GANSynth: adversarial neural audio synthesis. In: International conference on learning representations.

[ref27] Epley N. Waytz A. Cacioppo J. T. (2007). On seeing human: a three-factor theory of anthropomorphism. Psychol. Rev. 114, 864–886. doi: 10.1037/0033-295X.114.4.864, 17907867

[ref28] Epstein Z. Levine S. Rand D. G. Rahwan I. (2020). Who gets credit for AI-generated art? Iscience 23:101515. doi: 10.1016/j.isci.2020.101515, 32920489 PMC7492988

[ref29] Faiella A. Zielińska A. Karwowski M. Corazza G. E. (2025). Am I still creative? The effect of artificial intelligence on creative self-beliefs. J. Creat. Behav. 59:e70011. doi: 10.1002/jocb.70011

[ref30] Frazer J. G. (1959). The Golden Bough: A Study in magic and Religion. Macmillan Original work published 1890. Oxford, United Kingdom: Oxford University Press.

[ref6001] Frazer J. G. (1998). The golden bough: A study in magic and religion (Fraser R., Ed.). Oxford University Press. Oxford, United Kingdom. (Original work published 1890).

[ref9003] Furnham A. (1988). Lay theories: Everyday understanding of problems in the social sciences (1st ed.). Pergamon Press. Oxford, United Kingdom.

[ref31] Gangadharbatla H. (2022). The role of AI attribution knowledge in the evaluation of artwork. Empir. Stud. Arts 40, 125–142. doi: 10.1177/0276237421994697

[ref32] Gardner H. (1993). Multiple Intelligences: The Theory in Practice. New York, NY: Basic Books/Hachette Book Group.

[ref33] Gelan C. (2020). The concept of aura in Walter Benjamin's philosophy. Învăţământ, Cercetare, Creaţie 6, 115–128.

[ref34] Gelman S. A. (2003). The Essential Child: Origins of Essentialism in Everyday Thought. New York, NY: Oxford University Press.

[ref35] Grassini S. Koivisto M. (2024). Understanding how personality traits, experiences, and attitudes shape negative bias toward AI-generated artworks. Sci. Rep. 14:4113. doi: 10.1038/s41598-024-54294-4, 38374175 PMC10876601

[ref36] Gu S. Chen D. Bao J. Wen F. Zhang B. Chen D. . (2022). “Vector quantized diffusion model for text-to-image synthesis,” in Proceedings of the IEEE/CVF Conference on Computer Vision and Pattern Recognition, 10696–10706.

[ref37] Guthrie S. E. (1995). Faces in the Clouds: A New Theory of Religion. New York, NY: Oxford University Press.

[ref38] Heider F. Simmel M. (1944). An experimental study of apparent behavior. Am. J. Psychol. 57, 243–259. doi: 10.2307/1416950

[ref39] Hertzmann A. (2018). Can computers create art? Art 7:18. doi: 10.3390/arts7020018

[ref40] Hong J. W. Curran N. M. (2019). Artificial intelligence, artists, and art: attitudes toward artwork produced by humans vs. artificial intelligence. ACM Trans. Multimed. Comput. Commun. Appl. (TOMM) 15, 1–16. doi: 10.1145/3326337

[ref41] Hood B. (2009). Supersense: From Superstition to Religion-the Brain Science of Belief. London, United Kingdom: Hachette UK.

[ref42] Horton C. B. White M. W. Iyengar S. S. (2023). Will AI Art Devalue Human Creativity? Research Square. 10.21203/rs.3.rs-2987022/v1 (Accessed May 24, 2026).

[ref43] Jago A. S. (2019). Algorithms and authenticity. Acad. Manage. Discoveries 5, 38–56. doi: 10.5465/amd.2017.0002

[ref44] Javed M. A. Razzaq A. Khan T. Hussain M. Z. Hasan M. Z. Sarwar N. (2024). AI art generation threat or tool for traditional artists. Dial. Soc. Sci. Rev. (DSSR) 2, 685–704.

[ref45] Jung-Beeman M. Bowden E. M. Haberman J. Frymiare J. L. Arambel-Liu S. Greenblatt R. . (2004). Neural activity when people solve verbal problems with insight. PLoS Biol. 2:e97. doi: 10.1371/journal.pbio.0020097, 15094802 PMC387268

[ref6004] Kant I. (2000). Critique of the power of judgment (P. Guyer & E. Matthews, Eds. & Trans.). Cambridge University Press. Cambridge, United Kingdom. (Original work published 1790).

[ref48] Köbis N. Mossink L. D. (2021). Artificial intelligence versus Maya Angelou: experimental evidence that people cannot differentiate AI-generated from human-written poetry. Comput. Human Behav. 114:106553. doi: 10.1016/j.chb.2020.106553

[ref50] Kosmyna N. Hauptmann E. Yuan Y. T. Situ J. Liao X. H. Beresnitzky A. V. . (2025). Your Brain on ChatGPT: Accumulation of Cognitive Debt When Using an AI Assistant for Essay Writing Task, vol. 4. [Preprint]. arXiv. doi: 10.48550/arXiv.2506.08872

[ref51] Kounios J. Beeman M. (2014). The cognitive neuroscience of insight. Annu. Rev. Psychol. 65, 71–93. doi: 10.1146/annurev-psych-010213-115154, 24405359

[ref52] Kozbelt A. Dexter S. Dolese M. Seidel A. (2012). The aesthetics of software code: a quantitative exploration. Psychol. Aesthet. Creat. Arts 6:57. doi: 10.1037/a0025426

[ref53] Kruger J. Wirtz D. Van Boven L. Altermatt T. W. (2004). The effort heuristic. J. Exp. Soc. Psychol. 40, 91–98. doi: 10.1016/s0022-1031(03)00065-9

[ref54] Lulla M. Rajpurohit S. Vidani J. (2024). The Impact of AI on Creative Thinking Among Gen Z in Ahmedabad. Int. J. Sustain. Res., 2, 440–464. doi: 10.59890/1mndd531

[ref55] Magni F. Park J. Chao M. M. (2024). Humans as creativity gatekeepers: are we biased against AI creativity? J. Bus. Psychol. 39, 643–656. doi: 10.1007/s10869-023-09910-x

[ref6002] Mauss M. (2005). A general theory of magic. Routledge. London, United Kingdom. (Original work published 1902).

[ref57] Maynard P. (1997). The Engine of Visualization: Thinking Through Photography. Ithaca, NY: Cornell University Press.

[ref58] McCormack J. Gifford T. Hutchings P. (2019). “Autonomy, authenticity, authorship and intention in computer generated art,” in International Conference on Computational Intelligence in Music, Sound, Art and Design (Part of EvoStar), (Cham: Springer International Publishing), 35–50.

[ref59] Mednick S. (1962). The associative basis of the creative process. Psychol. Rev. 69, 220–232. doi: 10.1037/h0048850, 14472013

[ref60] Mikalonytė E. S. Kneer M. (2022). Can artificial intelligence make art?: folk intuitions as to whether AI-driven robots can be viewed as artists and produce art. ACM Trans. Hum. Rob. Interact. (THRI) 11, 1–19. doi: 10.1145/3530875

[ref61] Millet K. Buehler F. Du G. Kokkoris M. D. (2023). Defending humankind: anthropocentric bias in the appreciation of AI art. Comput. Hum. Behav. 143:107707. doi: 10.1016/j.chb.2023.107707

[ref62] Morris D. M. Alvey C. J. (2024). Knowledge and the perceived value of paintings: the role of time, presence, and the contagion effect on art evaluation. Psychol. Aesthet. Creat. Arts 18, 269–278. doi: 10.1037/aca0000464

[ref63] Most G. W. (2018). Hesiod: Theogony, Works and Days. Testimonia: Harvard University Press.

[ref64] Newman G. E. Bloom P. (2012). Art and authenticity: the importance of originals in judgments of value. J. Exp. Psychol. Gen. 141, 558–569. doi: 10.1037/a0026035, 22082113

[ref65] Newman G. E. Smith R. K. (2016). Kinds of authenticity. Philos. Compass 11, 609–618. doi: 10.1111/phc3.12343, 40046247

[ref66] Newman G. E. Smith R. K. (2018). “Artworks are evaluated as extensions of their creators,” in Advances in Experimental Philosophy of Aesthetics, In J. Robson (Ed.), Advances in experimental philosophy of aesthetics, London, United Kingdom: Bloomsbury Academic (pp. 103–121).

[ref67] Ostern N. K. Knickrehm C. Voss M. Kelch Y. (2024). Human-GAI Co-Creation: Using a Process Theory Lens to Understand Human-GAI Interaction in Creative Problem-Solving. ICIS 2024 Proceedings. International Conference on Information Systems (ICIS), Bangkok, Thailand. Available online at: https://aisel.aisnet.org/icis2024/user_behav/user_behav/6

[ref68] Park J. McGee R. (2023). Who, or rather, what painted this? Generation z’s attitudes towards artificial intelligence artworks. J. Stud. Res. 12, 1–14. doi: 10.47611/jsrhs.v12i4.5804

[ref69] Plucker J. A. Runco M. A. (1998). The death of creativity measurement has been greatly exaggerated: current issues, recent advances, and future directions in creativity assessment. Roeper Rev. 21, 36–39. doi: 10.1080/02783199809553924

[ref70] Ragot M. Martin N. Cojean S. (2020). “AI-generated vs. human artworks. A perception bias towards artificial intelligence?” in Extended Abstracts of the 2020 CHI Conference on Human Factors in Computing Systems, 1–10. doi: 10.1145/3334480.3382892

[ref72] Rozin P. Neneroff C. (2002). 11. Sympathetic Magical Thinking: The Contagion and Similarity “Heuristics”. In T. Gilovich, D. Griffin, & D. Kahneman (Eds.), Heuristics and Biases: The Psychology of Intuitive Judgment, Cambridge, United Kingdom: Cambridge University Press. 201–216.

[ref73] Scherer K. R. (2001). Appraisal considered as a process of multilevel sequential checking. Appraisal Processes in Emotion: Theory, Methods, Research, 92, 57,

[ref74] Shah A. (2023). How ChatGPT (AI) is likely to become a potential threat (or not) to human imagination and creativity? Int J Res Appl Sci Eng Technol (IJRASET) 11, 379–383. doi: 10.22214/ijraset.2023.55070

[ref75] Silvia P. J. Brown E. M. (2007). Anger, disgust, and the negative aesthetic emotions: expanding an appraisal model of aesthetic experience. Psychol. Aesthet. Creat. Arts 1, 100–106. doi: 10.1037/1931-3896.1.2.100

[ref76] Simonton D. K. (1988). Scientific Genius: A Psychology of Science. Cambridge, United Kingdom: Cambridge University Press.

[ref77] Simonton D. K. (1994). Greatness: Who Makes History and Why. New York, NY: Guilford Press.

[ref78] Simonton D. K. (2018). Defining creativity: don't we also need to define what is not creative? J. Creat. Behav. 52, 80–90. doi: 10.1002/jocb.137

[ref79] Takahashi S. (1995). Aesthetic properties of pictorial perception. Psychol. Rev. 102, 671–683. doi: 10.1037/0033-295X.102.4.671, 7480468

[ref81] Terzidis K. Fabrocini F. Lee H. (2023). Unintentional intentionality: art and design in the age of artificial intelligence. AI Soc. 38, 1715–1724. doi: 10.1007/s00146-021-01378-8

[ref82] Trach Y. (2021). Artificial intelligence as a tool for creating and analysing works of art. Cult. Arts Mod. World 22, 164–173. doi: 10.31866/2410-1915.22.2021.235907

[ref83] Trivigno F. V. (2020). Plato's Ion: Poetry, Expertise, and Inspiration. Cambridge, United Kingdom: Cambridge University Press doi: 10.1017/9781108581875.

[ref84] van Hees J. Grootswagers T. Quek G. L. Varlet M. (2025). Human perception of art in the age of artificial intelligence. Front. Psychol. 15:1497469. doi: 10.3389/fpsyg.2024.1497469, 39845559 PMC11750838

[ref85] Wade M. (2023). Is AI a threat to human creativity. Available online at: https://www.oxford-aiethics.ox.ac.uk/ai-threat-human-creativity (Accessed July 21, 2025).

[ref86] Wallas G. (1926). The Art of Thought. New York, NY: Harcourt, Brace.

[ref87] Weiner B. (1985). An attributional theory of achievement motivation and emotion. Psychol. Rev. 92, 548–573. doi: 10.1037/0033-295X.92.4.548, 3903815

[ref6003] Weisberg R. W. (2006). Creativity: Understanding innovation in problem solving, science, invention, and the arts. John Wiley & Sons, Inc. Hoboken, NJ.

[ref88] Wessell L. P. (1978). The aesthetics of living form in schiller and Marx. J. Aesthet. Art Crit. 37, 189–201. doi: 10.2307/429842

[ref89] Wiggins G. A. (2006). Searching for computational creativity. N. Gener. Comput. 24, 209–222. doi: 10.1007/bf03037332

[ref91] Wingström R. Hautala J. Lundman R. (2024). Redefining creativity in the era of AI? Perspectives of computer scientists and new media artists. Creat. Res. J. 36, 177–193.

[ref92] Zhou Y. Kawabata H. (2023). Eyes can tell: assessment of implicit attitudes toward AI art. i-Perception 14:20416695231209846. doi: 10.1177/20416695231209846, 38022746 PMC10663653

[ref93] Zylinska J. (2020). AI Art: Machine Visions and Warped Dreams. London, United Kingdom: Open Humanities Press.

